# 

**Published:** 1887-08

**Authors:** 


### BOOK NOTICES.

LIGHT, HEAT AND POWER, published at 413 Walnut St., Philadelphia, devoted especially to the *gas* industries and different illuminating methods, is hereafter to be issued bi-monthly. This speaks well for its prosperity and usefulness. We know of no better publication of its class in our language.

VICK'S ILLUSTRATED MONTHLY for July is a most excellent number, abounding in good things, as usual. No practical or amateur florist should fail to enrich himself by subscribing for it.

MEDICAL CLASSICS.—This is the name of a new medical journal issued every other month by the Medical Classics Publishing Co., 38 Murray St., New York City. The June number is largely devoted to the exposition, of the "tricks of trade" in medicines, particularly *quasi* medicated wines, beef extracts, etc. This is a broad field, and if even partially occupied, great good will come of it, not only to the sick and suffering, but to others who are accustomed to the use of questionable compounds.

THE CENTURY still maintains its place in the lead of our magazines. When all its contents are so admirable, it is unnecessary to particularise. There appears to be no abathment of interest in its war articles. As specimens of wood engraving, its illustrations are entitled to great praise.

"THERE'S NO ONE LIKE MOTHER TO ME." This is a new Song and Music, by Charles A. Davis, published by J. C. Greene & Co., 24 and 42 Arcade Street, Cincinnati. This is one of a series of original home songs to be published by the above well-known music house, and sent by mail to any address for 11 two cent stamps. The enterprise is a good one and should be encouraged by our music loving friends.

